# Daycase urology at Ibadan, Nigeria: a ten year review

**Published:** 2010-08-06

**Authors:** Takure Augustine Oghenewyin, Shittu Olayiwola Babatunde, Okeke Linus Ikechukwu, Olapade-Olaopa Oluwabunmi Emiola, Adebayo Sikiru Adekola

**Affiliations:** 1 Urology Division, University College Hospital (UCH), PMB 5116, Ibadan, Nigeria

**Keywords:** Day-case urology, day-case theatre, Ibadan

## Abstract

**Introduction:**

The objective of the study was to report the extent of urological surgeries performed as day cases in a major tertiary hospital in Ibadan and document the outcome.

**Methods:**

We retrospectively reviewed the data of all urologic patients treated at the day- case theatre between January 2000 and December 2009. The parameters evaluated were: age, diagnosis, type of surgery/ procedure, anaesthesia administered and the rank of the surgeon. Day case endoscopic procedures as well as prostate biopsies were excluded from the study.

**Results:**

In total, 1292 patients were managed, 290 (22.4%) were children aged between 5 days and 15 years while 1002 (77.6%) adults aged between 17 years and 91 years. The majority of the procedures were carried out by the senior registrars (1169 cases, 90.5%) and consultants were involved in 123 cases (9.5%). General anaesthesia was primarily used in children (158 children vs. 4 adults), while local anaesthesia was used only in adults. 131 (45.2%) children had no anaesthesia for circumcision. The most common procedures performed in adults were varicocelectomy (426 cases, 42.5%), orchidectomy (332 cases, 33.1%), testicular biopsy (120 cases, 12.0%). While in children male circumcision (231cases, 79.7%) was the main procedure performed.

**Conclusion:**

The day-case theatre is still suitable for day case urologic procedure in our setting. The common procedures are varicocelectomy, orchidectomy, and open testicular biopsy in the adults. While in children, male circumcision is commonly carried out.

## Introduction

Day case surgery (DCS) is the procedure carried out and the patient discharged on the same day. It was first documented by James Nicoll in Glasgow in 1909 and now well established in the United States of America, Europe, Australia, Asia and some part of Africa [1-3]. It is cost effective, significantly reduced waiting list and well tolerated by the patients 4. The increasing acceptability in both developed and developing world had led to its utilization by virtually all surgical specialties and more urologic patients are treated by DCS [4-8].

This retrospective study aimed to determine the extent of day case urology at the University College Hospital, Ibadan.

## Methods

We reviewed the records of urologic day cases performed in the division of urology over a ten year period (January 2000 to December 2009). We analysed the following data both for paediatric and adult patients admitted for urologic procedures: age, sex, indications for treatment, procedures performed, type of anaesthesia administered and the rank of the performing surgeon. We excluded endoscopic procedures such as internal urethrotomy, cystoscopy, bladder neck incision and ureteric catheterization because these procedures are done in the endoscopy outpatient theatre. Likewise prostate biopsy is not included because it is carried out in the surgery outpatient clinic. The data were collected in a proforma and analysed using SPSS version 17 using simple statistics.

## Results

During the study period, a total of 789 major elective urological cases were managed and 1292 day-case urological procedures treated in the daycase theatre, of which 1002 (77.6%) were adults aged between 17 and 91 years and children 290 (22.4%) aged between 5 days and 15 years.

All the urology day cases were males. The indication for surgery was therapeutic in 1166 (90.2%) patients and diagnostic in 126 (9.8%) patients. Tables 1 and 2 show the indications, number and type of day case procedures performed in adults and children respectively.

The most common procedures carried out in adults were varicocelectomy (n=426, 42.5%), orchidectomy (n=332, 33.1%) and open testicular biopsy (n=120, 12.0%) and others include hydrocelectomy (n=35, 3.5%), orchidopexy (n=30, 3.0%) and Herniorrhaphy (n=23, 2.3%). While male circumcision (n=231, 79.7%) was the most common procedure performed in children and others include re-do-circumcision (n=14, 4.8%), orchidopexy (n=14, 4.8%), urethral dilatation (n=14, 4.8%) and herniotomy (n=10, 3.5%).

General anaesthesia (GA) was primarily used in children (158 children vs. 4 adults), while local anaesthesia (LA) was used only in adults. 131 (45.2%) children with intact prepuce had no anaesthesia for circumcision.

The majority of the procedures were performed by the surgery residents (1169 cases, 90.5%), while the consultants managed 123 (9.5%) cases. There was no hospital admission.

In Ibadan the day case urologic procedures (DCU) are carried out in the day case theatre attached to the main theatre where major elective procedures are performed simultaneously. The patients are observed in the recovery room and are discharged home from here or in the side room attached to the main wards for the appropriate gender and age. This is in contrast to what obtains at Ile- Ife and Jos both in Nigeria where there are dedicated day surgery units as practiced in the developed world [2-4].

In addition, the closeness of the day case theatre to the main operating theatre allowed close supervision of the trainee surgical residents who performed 1169 (90.5%) of the day case urologic procedures and this finding is similar to other study in Nigeria [3]. It was responsible for no readmission of patients after these procedures.

DCU constituted 1292 (62%) of the total elective urologic procedures (1081) performed during our study period. However Sowande OA et al, found DCU as 51% comparable to UK and Australia while it was as low as 30.4% by Ojo E.O et al [3,4].

The scope of urologic procedures depends on the peculiarity of the environments as demonstrated in our study, where varicocelectomy, orchidopexy and testicular biopsy are the main procedures in adult. Male circumcision remained the most common procedure in children in Nigeria [3,4]. In previous studies diagnostic endoscopy and prostatic biopsy were more commonly carried out in the same theatre as open minor and major elective procedures. We excluded these procedures in our study because they were exclusively done at the surgery outpatient theatre [3-5,8]. Our findings becomes relevant because the volume of day cases done can encourage inter institution training of our trainee surgical residents so as to optimize their experiences in the training programme. We intend auditing our endoscopic day case procedures.

Unlike the previous studies in our environment, where all the circumcisions where performed without anaesthesia, our study, showed that 99 children had general and one caudal anaesthesia (16 weeks old child with intact prepuce) for circumcision. In the adults 990 (98.8%) tolerated local anaesthesia in form of 0.5% to 1% xylocaine with or without adrenaline [3,4].

**Table 1:** The indications and number of day case procedures/ surgery in adults

	Diagnosis	Type of procedure	number	%
Varicocele/ Oligoasthenoteratospermia	varicocelectomy	426	42.5%
Prostate cancer/undescended testes/testes mass	orchidectomy	332	33.1%
Infertility/testicular failure (primary/secondary)	open testicular biopsy	120	12.0%
Hydrocele	hydrocelectomy	35	3.5%
Horizontal lie testes/UDT	orchidopexy	30	3.0%
Hernia	herniorrhaphy	23	2.3%
Urethral stricture/post Urethroplasty stenosis	dilatation/rail roading (2)	12	1.3%
Prostate cancer+ hernia	orchidectomy/herniorrhaphy	07	0.7%
Epididymal cyst	excision	05	0.5%
Scrotal sebaceous cyst	excision	02	0.2%
Urethral warts	excision	03	0.3%
Bladder cancer	percutaneous biopsy	03	0.3%
Metastatic inguinal lymphadenopathy	lymph node biopsy	02	0.2%
Obstructive azoospermia	vasography	01	0.1%
Total		1002	100%

UDT: undescended testis

**Table 2:** The indications and number of day case procedures/surgery in children

	Diagnosis	Type of procedure	number	%
Intact prepuce/phimosis	Circumcision	231	79.7
Redundant prepuce/skin bridge	Re-do-circumcision	14	4.8
Undescended testis/horizontal testes	Orchidopexy	14	4.8
Urethral stricture/stenosis	Dilatation	14	4.8
Congenital hydrocele/hernia	Herniotomy	12	4.1
Meatal stenosis	Meatotomy	04	1.4
Post circumcision inclusion cyst	Excision	01	0.3
Total		290	100%

## Conclusion

We believe that the day case theatre is still suitable for day case urologic practice in our setting though a dedicated day care unit is preferred. In adults and children, open therapeutic procedures such as varicocelectomy, orchidectomy and circumcision were commonly performed while testicular biopsy was the main diagnostic procedure carried out in adults. There is the need for inter-institutional exchange programmes toward adequate surgical training.

## Competing interests

The authors declare they have no competing interests.

## Authors’ contributions

TAO: Concept and design, data collection and analysis, interpretation of data, initial draft, review of literature and final write up. Shittu OB: Design, data analysis, interpretation of data, critical review of initial draft and final write up. OLI: Critical review of draft and article and final write up. OEO: Critical review of article and final write up. ASA: Review of article and final write up.
